# 

**Published:** 1896-05-23

**Authors:** 


**Notices of Births, Deaths, and Marriages** are inserted in this column at a charge of 2s. 6d. for an announcement not exceeding four lines.

**Notice to Advertisers and Subscribers.**

All Advertisements, Orders for Copies of The Hospital and Business Communications should be addressed to **THE MANAGER, (Not to The Editor),** The Hospital, 428, Strand, London, W.C.

The Cost of Subscription, post free, is as follows:—Per week, 2½d.; per quarter, 2s. 8d.; per half-year, 5s. 3d.; per annum, 10s. 6d. Foreign:—Per Annum, 15s. 2d.; payable, in all cases, in advance.

Ready Shortly.

**"Burdett's Hospitals and Charities, 1896."**

Seventh year of publication. Over 1,000 pp. Scarlet cloth, gilt lettered, Price 5s. A most complete guide to British, Colonial, Indian, and American Hospitals, Dispensaries, Nursing and Convalescent Institutions, and Asylums. A few complete sets of the preceding six issues of the "Annual" may still be obtained on application to the Publishers, The Scientific Press (Limited), 428, Strand, London, W.C.

### NOTICE TO CORRESPONDENTS.

All MS., Letters, Books for Review, and other matters intended for the Editor should be addressed The Editor, The Lodge, Porchester Square, London, W.

The Editor cannot undertake to return rejected MS., even when accompanied by stamped directed envelope.

**NOTICE.—Vol. XIX. of "The Hospital" (October, 1895, to March, 1896), in handsome cloth binding, is now ready and on sale at the Office of this Journal. Price 7s. 6d. Cases for binding the Numbers contained in this Volume, 1s. 6d. Index to Vol. XIX., 2½d., post free.**

**The Monthly Part for May is now ready, price 6d., by post, 8d.**

### SCRAPS AND GLEANINGS.

IT is proposed to erect a new infectious hospital at Mousehole Lane, Southampton.

FIFTY-FIVE patients passed through the Ashby and District Cottage Hospital in 1895.

THE parishioners of Sedlescombe have protested against the proposed erection of the Battle Isolation Hospital.

THE Dunfermline Cottage Hospital is to be the recipient of a donation of £5,000 from the trustees of the late Earl of Moray.

HER Excellency the Countess Cadogan, accompanied by Lady Sophia Cadogan, visited Dr. Stevens's Hospital a short time ago.

THE Belfast Ophthalmic Institution and Eye and Ear Hospital treated 1,893 cases during the past year, an increase of 122 over 1894.

THERE is a movement on foot for the establishment of a convalescent home in connection with the North Riding Infirmary, Middlesbrough.

THE Ross Urban Council have convened a public meeting of the ratepayers to consider the need for providing a small infectious hospital for the district.

TWO HUNDRED AND FORTY-SEVEN in, and 1,961 out-patients were relieved in 1895 by the Oxford Eye Hospital, against 158 in and 1,744 out-patients in 1894.

THE Devon and Exeter Dental Hospital has decided to elect a representative of the Exeter Hospital Saturday Fund to a seat on the committee of the hospital.

AN infirmary, containing 120 beds, has been added to the Walsall Workhouse Buildings, the formal opening having taken place on March 31st. The cost has been about £8,000.

AT the close of the annual meeting of the Cardiff Provident Dispensary, recently held, the Secretary of the Metropolitan Provident Medical Association delivered a practical address on provident dispensary work.

THE new wing for infectious diseases at the Meath Hospital, Dublin, which was commenced in October, 1894, is now nearing completion. When opened 40 more beds will be added to the 116 beds it at present contains.

THE managers of the Victoria Hospital for Sick Children, Hull, have commenced to put aside an endowment fund, to which all legacies and special donations will in future be paid. The present amount of the fund is £1,750.

A WARD at the Rotunda Hospital at Dublin has been named the "Eleanor C. and William Josiah Smyly Ward," in recognition of the services rendered to the hospital by the master, Dr. Smyly, and by Mr. W. J. Smyly.

THE annual report of the Worcester Dispensary shows that 1,443 applications to join the provident branch were made during 1895 as compared with 1,427 in the preceding year. The total number of members amounted to 9,299.

THE number of cases attended by the Birmingham Lying-in Charity in 1895 was 798, a considerable falling-off compared with previous years, owing probably, it is said, to an improved state of trade. The cost per head of attending these cases is given in the report as 13s. 2¾d.

THERE has been a slight increase in the annual subscriptions received by the Victoria Eye and Ear Hospital, Hereford, £367 having been received from this source in 1895, against £339 in 1894. Donations and church collections also show an increase. Twelve hundred cases were treated during the past year, of which 91 were in-patients.

THE number of new members registered at the Hockley Branch Dispensary in 1895 was 308, increasing the total roll to about 1,600. This dispensary was originally founded as a purely charitable dispensary by the late Mr. Sands Cox, but, the funds left for its support being insufficient, it has, to a great extent, been transformed into a provident institution.

THE average weekly cost of each inmate (including servants) of the Moseley Hall Convalescent Home is stated to have been 5s. 0½d. in 1895, as against 4s. 0¾d. in 1894. The home was closed for five months during the year, owing to much needed sanitary alterations having to be undertaken. These cost £3,600, and in consequence, the accounts for 1895 show a deficiency of over £1,500.

A PROPOSAL has been made to establish a maternity hospital in connection with the Dundee Infirmary. The House Committee wisely require a guarantee, before they undertake this work, that the sum necessary for maintenance will be forthcoming. They calculate that £10,000 is necessary to erect and furnish a suitable building, and to provide about one half of the £500 a year which it is estimated the working of the maternity hospital will cost.

THE directors of the Paisley Infirmary had decided, owing to the cost being considerably more than was anticipated, to leave out for the present the third or west pavilion, with its corresponding corridor, of the new infirmary which is being erected. Recently, however, a gentleman who has taken a great interest in the institution has intimated that a sufficient amount of money has been guaranteed to him to enable the whole of the scheme to be put in hand.

THE clergy and ministers of all denominations of Ilkley and the neighbourhood might surely do better for the Ilkley Hospital and Convalescent Home than they did last year. Only one congregation—the Wesleyan Chapel—devoted a collection to the hospital. According to the report the average cost per patient was £1 13s. 2½d. in 1895, against £1 13s 2d. in 1894, of which sum household expenses amounted to 19s. and 18s. 11d. respectively. The receipts for 1895 amounted to £2,003, and the actual expenditure to £ ,753.

IN order to encourage patients presenting themselves for free relief (either with a subscriber's letter or on an infirmary order) to become provident members, the committee of the Sunderland Provident Dispensary last year tried the experiment of paying the admission fees out of a fund specially allocated to this purpose. The experiment was continued for about six months, during which time 563 cases were assisted in this way, but, unfortunately, it was found that only about one-fourth of the number evinced any desire to make a further payment, with the result that, except in a few cases, the committee have now ceased the practice.

### THE STUDENT OF MEDICINE.

### VACANCIES.

### Resident Medical Officers.

Bridgwater Infirmary.—House Surgeon. Salary £80 per annum, with board and residence. Applications to Mr John Coombs, Honorary Secretary, Bridgwater Infirmary, Bridgwater, by May 29th.

City of Birmingham.—Medical Superintendent for the City Hospital for Infectious Diseases, Little Bromwich, Birmingham. Must be under 40 years of age, unmarried, doubly qualified. Salary £200 per annum, rising £20 yearly to £300 per annum, with apartments, rations, and attendance. Applications, endorsed "Medical Superintendent," to be addressed to Mr. J. Keete, Clerk to the Health Committee, Council House, Birmingham, by May 29th.

Horton Infirmary, Banbury.—House Surgeon and Dispenser. Salary £60 per annum, with board and lodging. Applications to Mr. O. H. Davids, Honorary Secretary, 21, Marlborough Road, Banbury, by May 23rd.

Hospital for Consumption and Diseases of the Chest, Brompton.—Resident Assistant Medical Officer. Salary £50 per annum, with board and residence. Applications to the Secretary by May 27th.

Hospital for Sick Children, Great Ormond Street, Bloomsbury.—House Surgeon; unmarried. Appointment for six months. Salary £20, with board and residence in the hospital. Resident Medical Superintendent. Appointment for one year subject to re-election. Salary £105 per annum, with board and residence in the hospital. Applications, on forms to be provided, to be sent to the Secretary by May 29th.

National Hospital for Diseases of the Heart and Paralysis, Soho Square.—Resident Medical Officer; doubly qualified. Board, residence, laundry, and an honorarium of 10 guineas for the term of office for six months. Applications to the Secretary by May 25th.

Royal Hospital for Diseases of the Chest, City Road.—House Physician. Appointment for six months. Salary at the rate of £40 per annum, with lodging and laundry. Applications to the Secretary by May 25th.

Stockport Infirmary.—Junior Assistant House Surgeon; doubly qualified. Appointment for six months, with board and residence. An honorarium of £10 given after six months' satisfactory service. Applications to the Secretary by May 26th.

Weston-super-Mare Hospital and Dispensary.—Medical Officer to the Provident Dispensary; doubly qualified. Salary £80 per annum, with board, lodging, and washing. Applications to the Honorary Secretary by May 26th.

### Non-Resident Medical Officers.

Hospital for Women, Soho Square, W.—Assistant House Physician. Appointment for three months. Applications to David Cannon, Secretary, by May 26th.

Paddington Green Children's Hospital, London, W.—Physician to Out-patients, and Surgeon to Out-patients. Applications to the Secretary by May 30th.

Royal College of Surgeons of England.—Hunterian Professors, Erasmus Wilson Lecturer, and Arris and Gale Lecturer for the ensuing years. Applications to Edward Trimmer, Secretary, by June 2nd.

**WESTON-SUPER-MARE HOSPITAL AND DISPENSARY.**

